# Influenza infection and heart failure—vaccination may change heart failure prognosis?

**DOI:** 10.1007/s10741-017-9614-7

**Published:** 2017-05-15

**Authors:** Nikolaos P. E. Kadoglou, Frank Bracke, Tim Simmers, Sotirios Tsiodras, John Parissis

**Affiliations:** 1 Department of Cardiology, Catharina Hospital, Michelangelolaan 2, 5623 EJ Eindhoven, The Netherlands; 2 Centre for Statistics in Medicine—Βotnar Research Centre, University of Oxford, Oxford, UK; 3 4th Department of Internal Medicine, Attikon University Hospital, Athens, Greece; 4 Heart Failure Unit, Department of Cardiology, Attikon University Hospital, Athens, Greece

**Keywords:** Influenza infection, Heart failure, Decompensation, Mortality, Hospitalization, Dose, Vaccination, Immunization

## Abstract

The interaction of influenza infection with the pathogenesis of acute heart failure (AHF) and the worsening of chronic heart failure (CHF) is rather complex. The deleterious effects of influenza infection on AHF/CHF can be attenuated by specific immunization. Our review aimed to summarize the efficacy, effectiveness, safety, and dosage of anti-influenza vaccination in HF. In this literature review, we searched MEDLINE and EMBASE from January 1st 1966 to December 31st, 2016, for studies examining the association between AHF/CHF, influenza infections, and anti-influenza immunizations. We used broad criteria to increase the sensitivity of the search. HF was a prerequisite for our search. The search fields used included “heart failure,” “vaccination,” “influenza,” “immunization” along with variants of these terms. No restrictions on the type of study design were applied. The most common clinical scenario is exacerbation of pre-existing CHF by influenza infection. Scarce evidence supports a potential positive association of influenza infection with AHF. Vaccinated patients with pre-existing CHF have reduced all-cause morbidity and mortality, but effects are not consistently documented. Immunization with higher antigen quantity may confer additional protection, but such aggressive approach has not been generally advocated. Further studies are needed to delineate the role of influenza infection on AHF/CHF pathogenesis and maintenance. Annual anti-influenza vaccination appears to be an effective measure for secondary prevention in HF. Better immunization strategies and more efficacious vaccines are urgently necessary.

## Influenza infection and heart failure

### Influenza infection burden

Influenza is a respiratory RNA virus with two types, A and B, causing epidemic human disease in a seasonal pattern [1]. Based on surface antigens (e.g., hemagglutinin, neuraminidase), influenza virus type A is further classified into subtypes [2]. Immunity to surface antigens protects individuals against infection from one influenza virus type or subtype but confers little or no immunity against other types and subtypes. Frequent mutations in haemagglutinin and neuramidase antigens (antigenic drift) create new variants circumventing existing immunity in a partial or complete way. In cases of antigenic drift influenza strains are more likely to cause seasonal epidemics [3] with limited coverage by circulating vaccines prepared well in advance from the annual seasonal epidemic.

In the absence of a global anti-influenza vaccine conferring permanent immunity, influenza remains one of the leading causes of widespread viral diseases, with high morbidity and mortality. An estimated 5–10% of adults and 20–30% of children are infected by influenza worldwide, while 250,000–500,000 severely infected individuals are estimated to die annually [4].

The association between influenza and cardiovascular morbidity and mortality is confounded by several factors. Firstly, significant under-testing and under-diagnosis occurs in affected patients. Point-of-care molecular testing is becoming gradually widely available with emphasis on specificity during the influenza season and on sensitivity outside it [5]. Secondly, influenza infections impact may vary according to virus type, duration of circulation, the prevalence of “high risk” population groups, including the elderly and patients with other besides cardiac disease, chronic co-morbidities (e.g., chronic lung disease, diabetes mellitus, cancer, etc.) and underlying herd immunity/immunization defects [6].Thirdly, most influenza-related deaths are frequently under-reported, because influenza often exacerbates underlying diseases, i.e., cardiac disease that may be recorded as the primary cause of death [7]. Thus, the number of influenza-related deaths is often monitored as the number of excess deaths compared to a period without known influenza viruses’ activity [8]. Similarly, there is increased occurrence of bacterial co-infections, i.e., pneumonia, meningitis, in the course of influenza infection [9], overlapping the influenza viruses’ impact.

Thus, a wide-spectrum of influenza-related consequences can be seen in patients with chronic disease including cardiac patients ranging from uncomplicated recover within 1 week of illness without requiring any medical intervention to a severely ill patient requiring intensive care unit treatment. Statistical modeling methods and health system records have estimated a high annual burden of influenza-attributable hospitalizations and deaths resulting in a significant strain on national health systems and substantial economic loss [10].

### Methods of literature search

The aim of the present clinical review was to evaluate the literature about the efficacy, effectiveness, safety, and dosage of anti-influenza vaccination in HF patients. A search was conducted for English language publications in MEDLINE and EMBASE databases from January 1st 1966 to December 31st, 2016. The following broad search terms, including Medical Subject Headings (MeSH), were used: chronic heart failure (CHF), acute heart failure (AHF), acute decompensated heart failure (ADHF), influenza infection, vaccination, immunization, hospitalizations, mortality, death, and heart failure decompensation. Two investigators (NK and ST) performed the literature research. With the exception of case studies, all other study designs were considered (both randomized and non-randomized), whether prospective or retrospective. We excluded studies that did not report heart failure outcomes. The reference list of the included articles was checked to identify other relevant papers for inclusion.

### Interaction of influenza infection with acute and chronic heart failure development

Numerous observational studies have shown an elevated risk for acute cardiovascular events (e.g., stroke, acute myocardial infarction—AMI,) in general population, not only within the first few days of infection [11–13], but also in the long term (>30 days) [11].

#### Influenza infection and development of chronic heart failure

**Fig. 1:**
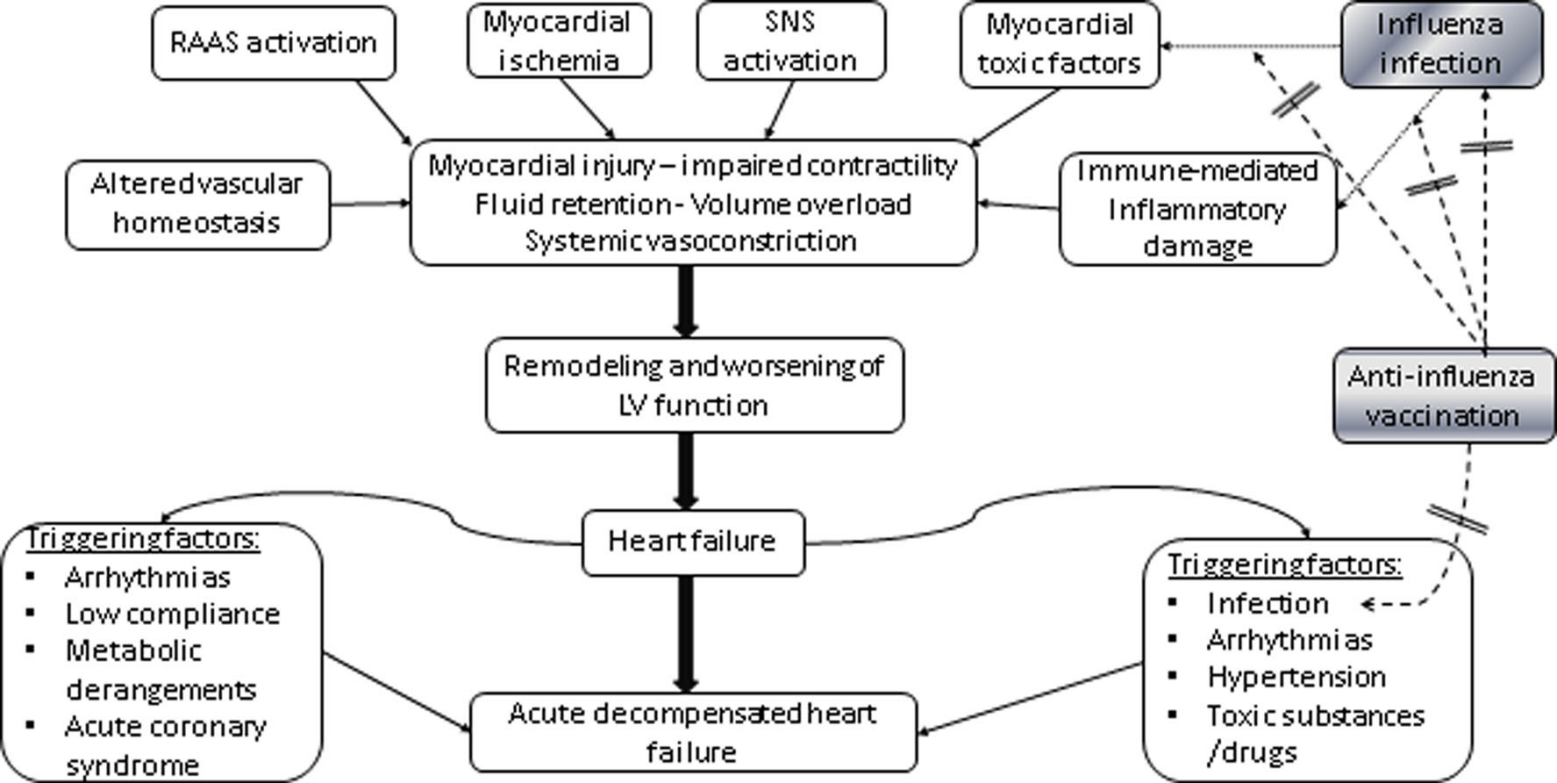
Pathophysiologic mechanisms of chronic heart failure development and its acute decompensation. The potential preventive effect of anti-influenza vaccination. *RAAS* renin-angiotensin-aldosterone system; *SNS* sympathetic nervous system, *LV* left ventricle

It is still obscure whether influenza infection can be a primary cause of heart failure (HF). A wealth of information implicates the involvement of inflammatory activation in the development and progression of CHF [14]. Nevertheless, a pure causative association between infection and both AHF and CHF development is still elusive. Influenza infection may induce acute, direct myocardial dysfunction through the stimulation of immune system and inflammation [15, 16]. This derives from histologically evident direct myocardial injury, myocarditis, and myocyte necrosis (found in myocardial tissue samples after influenza-related deaths). In addition to this, the high metabolic demands, and potent inflammatory and thrombotic agents activated by infection may indirectly suppress myocardial function leading to either new onset HF or acute decompensation of chronic HF (see below). Pro-inflammatory cytokines release, endothelial dysfunction, and sympathetic activation comprise the common pathophysiologic mechanisms of HF [14]. Influenza accompanied by changes in cardio-renal function may exaggerate fluids shift, leading to volume overload and hence HF development, progression, or decompensation [17, 18]. Unfortunately, all the above mechanistic explanations are still speculative and to our knowledge, no studies have ever shed light on the pathophysiologic interference between new onset HF and influenza infection, as well as the potential protective effect of anti-influenza vaccination **(**Fig. 1 **)**.

#### Influenza infection and development of acute decompensated heart failure (ADHF)

Infection-induced ADHF may develop on the top of pre-existing HF, already diagnosed or not [19, 20]. A significant indication of the interaction between ADHF and influenza comes from the parallel fluctuations of HF-related hospitalizations and seasonal infections like influenza [21]. Indeed, long-term monitoring of USA population showed the peak of hospitalizations due to ADHF during winter and the lowest curve during summer. The seasonal variation of ADHF-related morbidity and mortality has been also mentioned in numerous, national-based cohort studies [21–23]. This is reasonable since patients with HF appear with limited cardiac and respiratory reserves are older and unable to tolerate infection-induced cardiac compromise. In those frail patients with CHF, the elaboration of pro-inflammatory and oxidative mediators may easily lead to ADHF [24]. Moreover, the pre-existed vascular congestion may mechanically induce endothelial activation triggering an acute decompensation [25, 26]. On the other hand, influenza infection highly predisposes to stimulation of all the latter mechanisms. Thereby, the co-existence of HF and influenza may easily lead to the detrimental ADHF [11, 21].

## Anti-influenza vaccination and heart failure

### Anti-influenza vaccination and cardiac diseases

Immunization against seasonal influenza has risen during the last decades as a potential measure to reduce hospitalization rate [27, 28] and all-cause mortality among elderly subjects [29–31] during influenza epidemics. Community-based studies support the preventive effectiveness of anti-influenza vaccination on cardiovascular outcomes, but their results are not consistent. A recent systematic review [13] and most [32, 33], but not all [34], observational studies favor the timely implementation of anti-influenza vaccination to prevent AMI incidence. In addition to primary prevention, anti-influenza vaccination has been reported as an effective preventive measure in patients with already established CAD [35]. Other researchers have argued that there is little or even no effect of anti-influenza vaccination on cardiovascular diseases-related hospital admissions [36, 37]. According to them, vaccination favorable effect on hospitalization rate is modest and only confined in the prevention of respiratory infection. From the vaccine perspective, selection bias, variation of influenza seasons’ duration and unmeasured confounders might have affected the final results.

Regarding those controversies, the largest international scientific societies have considered the magnitude of data as robust evidence of annual anti-influenza vaccination for secondary, but not primary, prevention in patients with cardiovascular diseases, like those with CAD or HF [38, 39]. As a secondary prevention measure, anti-influenza vaccination may be considered in type 2 diabetic patients with a higher predisposition to cardiovascular diseases [40]. Notably, admissions for HF were significantly reduced in the flu season in vaccine recipients compared to those who did not receive the vaccine. Focusing on patients with HF, anti-influenza vaccination has the potential to modify the pathophysiologic mechanisms of HF (Fig. 1). However, current data supporting the pre-emptive implementation of anti-influenza vaccination to primarily prevent the de novo CHF development are missing.

### Anti-influenza vaccination in patients with pre-existed heart failure

The advocacy of anti-influenza vaccination in HF is based on limited number of large, observational studies. There are additional data derived from population-based registries (Table 1). Methodological issues prevent an adequate response to the question of how effectively vaccination does prevent ADHF. Some studies have recorded higher hospitalization rates [46] and longer hospital stays [17] in unvaccinated patients with HF exacerbations. Those effects were more pronounced among elderly and patients with chronic kidney disease [47]. Despite the clinical relevance of those findings, they are based on AHF reports as reasons of hospital admission, without examining quantitative data about myocardial function prior to the event.

**Table 1:** Summarized list of studies concerning the effects of influenza vaccination on clinical outcomes in patients with pre-existed heart failure

Source	Study design/number of subjects	Vaccination/follow-up	Results of vaccination
De Diego C et al. (2009) [31]	Observational, community-based study/1340 Spanish pts., ≥65-year-olds with chronic heart disease (congestive HF or CAD)	480 annually vaccinated pts. vs. 860 non-vaccinated pts. FU: 40 months	↓All-cause mortality throughout the overall influenza periods (January–April) compared to the reference non-influenza periods (June–September) (adjusted HR: 0.63; 95% CI: 0.44–0.91; *p* = 0.013) ↔All-cause mortality during the reference non-influenza period (adjusted HR: 0.94; 95% CI: 0.56–1.58; *p* = 0.814).
Liu IF et al. (2012) [41]	Observational population-based study/5048 Taiwanese elderly pts. with ischemic heart disease (HF, past MI, CAD)	2760 vaccinated pts. vs. 2288 non-vaccinated pts. FU: 4 years	During influenza season: ↓All-cause mortality (HR: 0.42; 95% CI: 0.35–0.49) ↓Hospitalization for CVD (HR: 0.84; 95% CI: 0.76–0.93) In the whole year: ↓All-cause mortality (HR: 0.78; 95% CI: 0.68–0.90)
Mohseni H et al. (2016) [42]	Self-controlled case series from the primary care database in UK/59,202 HF pts.	Within person comparison: an influenza vaccination in any subsequent year vs. an adjacent vaccination-free year (before or after). FU: 2 consecutive years	↓Hospitalizations due to CVD (incident rate ratio: 0.73; 95%CI: 0.71–0.76) ↓Hospitalizations due to all-causes (incident rate ratio: 0.96; 95%CI: 0.95–0.98)
Kopel E et al. (2014) [43]	Heart Failure Survey in Israel (HFSIS), prospective study/1964 pts. hospitalized with acute HF	501 vaccinated pts. vs. 1453 non-vaccinated pts. FU: End of each hospitalization, 1 and 4 years	↓1-year all-cause mortality (HR: 0.81; 95%CI: 0.66–0.99, *p* = 0.004) ↓4-year all-cause mortality (HR: 0.83; 95%CI: 0.73–0.95, *p* = 0.006)
PARADIGM-HF (2016) [44]	Sub-analysis of PARADIGM-HF trial: randomly allocated to sacubitril/valsartan or enalapril/8399 symptomatic, HF pts. with reduced EF (<40%)	1769 vaccinated pts. vs. 6630 non-vaccinated pts. Vaccination 12 months prior to study entrance. FU: 12 months post-randomization	↓All-cause mortality (HR: 0.81; 95% CI: 0.67–0.97; *p* = 0.015). ↑Rates of cardiopulmonary, influenza-related, and all-cause hospitalization in unadjusted models, but not in propensity-adjusted models
Wu WC et al. (2014) [45]	Retrospective analysis/ 2739 HF pts. treated in Veterans Health Administration Hospitals	2087 vaccinated pts. (before or during admission) vs. 429 non-vaccinated pts. FU: up to 1 year	↓30-day adjusted all-cause mortality (OR: 0.49; 95%CI: 0.29–0.83) ↓1-year adjusted all-cause mortality (OR: 0.74; 95%CI: 0.58–0.96)

*RAAS* renin-angiotensin-aldosterone system; *SNS* sympathetic nervous system, *LV* left ventricle, *HR* hazard ratio, *FU* follow-up, *yr* years, *CI* confidence interval, *CVD* cardiovascular disease, *CAD* coronary artery disease, *HF* heart failure, *pts.* patients, *OR* odd ratio, *EF* ejection fraction, *SOLVD* Studies of Left Ventricular Dysfunction,

To address that question, a previous observational, population-based study examined the clinical impact of anti-influenza vaccination on patients with diagnosis of CAD and/or ischemic HF [41]. There was an independent association of vaccination with reduced risk of heart disease-related hospitalizations only during influenza seasons. Another community-based study demonstrated reduced all-cause mortality only throughout the influenza periods (January–April) in four consecutive years in Spanish patients with congestive HF or CAD who received annual anti-influenza vaccination [31]. Most recently, Mohseni et al. (2017) [42], using a self-controlled case series design, demonstrated the association of anti-influenza vaccination with reduced hospitalization risk, especially for cardiovascular diseases, in 59,202 HF patients. Additional data underpinned reduced hospitalization rate and survival benefits, not only during influenza seasons, but persistently in the whole year in vaccinated HF patients [39, 43]. Although the favorable results of vaccination were independent of several confounders, none of the above studies was powered to prove cause-effect relationship between vaccination and survival. Presumably, unmeasured confounding factors and bias, like the co-existence of higher medical surveillance and socioeconomic status in vaccinated patients, may explain their improved survival.

A sub-analysis of the large-scale PARADIGM-HF trial was recently published [44]. The main aim of PARADIGM-HF trial was the comparative evaluation of LCZ696 (sacubitril/valsartan) and enalapril in patients with symptomatic HF and reduced EF (<40%). That sub-analysis revealed lower risk for all-cause mortality in vaccine recipients compared to the non-vaccinated group. Although influenza vaccination did not affect the superior clinical benefits of LCZ696 over enalapril during the follow-up period (median duration: 27 months), vaccine remained an independent determinant of survival in the propensity-adjusted models. A negative effect of vaccine implementation in that trial was the higher risk for all-cause hospitalization. However, that risk was blunted after propensity adjustment, implicating common confounders. All the above results should be cautiously evaluated to extrapolate conclusions [48]. Firstly, the published sub-analysis collected data from the PARADIGM-HF trial database that was not originally designed to assess the effectiveness of anti-influenza vaccination; thus residual confounding is a major issue in proving direct vaccination effects. Secondly, it was a multi-center, international trial recruiting patients from different health systems with diverse compliance to guidelines. That was depicted by the wide variety of vaccination rates along different countries (from 0 to 77.5%), leading to an extremely low overall prescription rate of vaccine (21%). In developed countries the vaccination rate was higher, probably because their residents had easier access to high quality healthcare services. The latter phenomenon could explain either the higher trend for hospitalization or the reduced mortality observed in vaccinated group. On the other hand, unvaccinated patients mostly originated from undeveloped countries and used life-saving therapeutic modalities (e.g., b-blockers, ICDs etc) at a much lower frequency; this might have influenced the final results.

In terms of effectiveness, anti-influenza vaccination seems to reduce HF-related hospitalizations and all-cause mortality, especially during influenza periods, in patients with pre-existed HF.

### High dose versus standard dose of vaccination in heart failure

The vaccine effectiveness relies upon intact immune responses, which may be compromised in HF. Following influenza immunization most individuals develop both antibody titers and T-cell immune responses. The efficacy (laboratory measured) and consequently the effectiveness (clinical outcomes) of anti-influenza vaccine seem to decrease with increasing age and the presence of co-morbidities [49]. Evidence derived from small previous studies in HF patients suggests waning humoral-mediated [50] and T-cell [51] immunity response to vaccination compared to healthy controls. Albrecht CM et al. (2014) [52] observed a significant decline of mean antibody titers to both the A/H3N2 and A/H1N1 influenza strains over a 12-month post-vaccination period in HF patients compared to healthy controls.

A more aggressive approach of vaccination using high rather than standard-dose vaccine in elderly patients has been proposed [53]. Notably, a recently published randomized, double-blind, active-controlled efficacy trial in 31,989 medically stable adults aged >65 years, documented the superiority of four-fold dose over standard dose in reducing the incidence of influenza caused by any viral type or subtype [49]. In the same study, which included patients with stable HF and/or CAD, high-dose vaccine recipients showed lower rates for pneumonia, cardiorespiratory disturbances, and medications prescription. All those clinical data were not comparatively evaluated with anti-bodies titers. A small, pilot, randomized, double-blind, active-controlled study investigated the double dose versus standard dose of influenza vaccination among 28 patients with HF [54]. The double dose group appeared with more vigorous (within 2–4 weeks post-vaccination) response with higher antibody titers to A/H3N2, A/H1N1, and B-type influenza antigens. The absolute antibody titers remained above seroprotective levels, but the difference between groups was eliminated after 4–6 months. The authors commented the higher initial antibody titers as a promising sign of higher degree protection against influenza infection. However, these preliminary data were based on a small sample and the clinical relevance of antibody titers alterations is doubtful [55]. Concerning the reduced immune response in patients with even mild HF, unambiguously, further large studies are necessary to draw firm conclusions about the appropriate and safe dose of anti-influenza vaccine provided that no substantial drift exists.

### Anti-influenza vaccination safety and timing in heart failure patients

Despite the previously mentioned overwhelming evidence of the burden of influenza and the high effectiveness of anti-influenza vaccine, the fear of negative side effects has discouraged many people from getting vaccinated. Evidence deriving from the general population has failed to show an association between influenza vaccines and significant side effects [56, 57]. Given the acceptable safety profile of influenza vaccines and the World Health Organization’s recommendation for its use in high-risk populations, influenza immunization seems to be a cost-effective and safe approach to decrease influenza burden in HF.

Influenza vaccines confer only short-lived, strain-specific immunity, and annual revaccination is required. Optimal timing is difficult to predict, since the influenza season starts at different times each year and influenza activity may vary among geographic regions. In clinical practice, the administration of anti-influenza vaccine in HF patients is wise to take place in pre-influenza seasons (prior to winter), with renewed formulation each time. Interestingly, a large retrospective study in male veterans admitted to hospitals due to HF underpinned the survival benefits of anti-influenza vaccination not only among patients vaccinated before, but also during hospitalization [45]. Thereby, the authors recommended vaccination of HF patients even as part of in-hospital practice settings.

## Future implication of vaccination in heart failure

The majority of large studies have enrolled community-dwelling populations with mixed characteristics (e.g., heart or lung diseases), where it is difficult to distinguish HF patients. Moreover, HF is mostly referred as the admission reason for patients with concomitant influenza infection, while only a limited number of studies have clearly defined cohorts with pre-existed HF. Hence, the future research should point towards populations with established HF. Details of pre- and post-infection clinical characteristics and medications records are necessary in order to retrieve the pure effect of anti-influenza vaccination on HF progression and precisely assess the incidence of HF decompensation. It would be also prudent to evaluate the vaccination effects along the classes of HF severity. Another important drawback of observational studies and meta-analyses is the difficulties to evaluate classify a death as influenza-related mortality. For this purpose, most researchers have presented results on all-cause mortality instead of specific-cause mortality. Therefore, a residual confounding in the estimates of vaccine effectiveness cannot be entirely excluded.

According to prevailing recommendations, the anti-influenza vaccination should be encouraged in HF patients, who are at high mortality risk. Unfortunately, they often have suboptimum vaccine uptake. A significant proportion of elderly people with chronic HF remain annually unvaccinated against influenza [31, 44]. A plethora of factors like beliefs, attitudes and opinions of patients and health professionals, socioeconomic and educational conditions, race minorities, and vaccination during previous years may act as obstacles to sustain vaccination programs. Another important determinant of vaccination implementation is the management of healthcare systems. Only well-organized national and international campaigns, based on robust evidence, may boost vaccination in routine practice. Most European countries still remain well below the 75% proposed coverage threshold for people older than 65 years or patients with high-risk conditions.

Large-scale and adequately powered studies targeting the effectiveness and safety of anti-influenza vaccination will assess beyond any doubt the cost-effectiveness of anti-influenza vaccination in HF patients (secondary prevention) or those at increased risk for HF development (primary prevention).

## Conclusion

Seasonal influenza infection, especially among elderly populations, is associated with high occurrence of AHF, increased cardiovascular morbidity and all-cause mortality. Based on data from the general population, annual anti-influenza vaccination seems to be an effective measure to prevent AHF episodes and in some degree to improve survival. Confining literature research in patients with pre-existed HF, there are limited data favoring the incorporation of anti-influenza vaccination as an adjunctive therapy, to reduce hospitalizations and all-cause mortality rates. Unambiguously, future studies will verify the required dose and the potential benefits and risks of annual anti-influenza vaccination in the context of primary prevention or standard care processes of established HF (like medications, LV function assessment, etc).
